# Clinical significance, molecular characterization, and immune microenvironment analysis of coagulation‐related genes in clear cell renal cell carcinoma

**DOI:** 10.1002/cai2.105

**Published:** 2024-01-07

**Authors:** Weihao Chen, Xupeng Zhao, Yongliang Lu, Hanfeng Wang, Xiyou Wang, Yi Wang, Chen Liang, Zhuomin Jia, Wei Ma

**Affiliations:** ^1^ Department of Urology The Third Medical Center of PLA General Hospital Beijing China; ^2^ School of Medicine Nankai University Tianjin China; ^3^ Medical Service Department The PLA General Hospital Beijing China; ^4^ Senior Department of Otolaryngology‐Head & Neck Surgery The Sixth Medical Center of PLA General Hospital Beijing China

**Keywords:** clear cell renal cell carcinoma, coagulation, immunotherapy, risk score, tumor immune microenvironment

## Abstract

**Background:**

Numerous studies have revealed a tight connection between tumor development and the coagulation system. However, the effects of coagulation on the prognosis and tumor microenvironment (TME) of clear cell renal cell carcinoma (ccRCC) remain poorly understood.

**Methods:**

We employed the consensus clustering method to characterize distinct molecular subtypes associated with coagulation patterns. Subsequently, we examined variations in the overall survival (OS), genomic profiles, and TME characteristics between these subtypes. To develop a prognostic coagulation‐related risk score (CRRS) model, we utilized the least absolute shrinkage and selection operator Cox regression and stepwise multivariate Cox regression analyses. We also created a nomogram to aid in the clinical application of the risk score, evaluating the relationships between the CRRS and the immune microenvironment, responsiveness to immunotherapy, and targeted treatment. The clinical significance of PLAUR and its biological function in ccRCC were also further analyzed.

**Results:**

There were significant differences in clinical features, prognostic stratification, genomic variation, and TME characteristics between the two coagulation‐related subtypes. We established and validated a CRRS using six coagulation‐related genes that can be employed as an effective indicator of risk stratification and prognosis estimation for ccRCC patients. Significant variations in survival outcomes were observed between the high‐ and low‐risk groups. The nomogram was proficient in predicting the 1‐, 3‐, and 5‐year OS. Additionally, the CRRS emerged as a novel tool for evaluating the clinical effectiveness of immunotherapy and targeted treatments in ccRCC. Moreover, we confirmed upregulated PLAUR expression in ccRCC samples that was significantly correlated with poor patient prognosis. PLAUR knockdown notably inhibited ccRCC cell proliferation and migration.

**Conclusion:**

Our data suggested that CRRS may be employed as a reliable predictive biomarker that can provide therapeutic benefits for immunotherapy and targeted therapy in ccRCC.

## Graphical abstract

This study found that coagulation subtypes were associated with prognosis and immune microenvironment in clear cell renal cell carcinoma patients. A precise prognostic coagulation‐related risk score (CRRS) model was successfully established. The CRRS model revealed a potential prognostic value for the immunotherapy response.

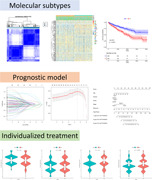

**Abbreviations**
AUC: area under the curve
ccRCC: clear cell renal cell carcinoma
CRGs: coagulation‐related genes
CRRS: coagulation‐related risk score
FBS: fetal bovine serum
GEO: Gene Expression Omnibus
GSVA: gene set variation analysis
IC50: semi‐inhibitory concentrations
IHC: Immunohistochemistry
IPS: immunophenoscore
KEGG: Kyoto Encyclopedia of Genes and Genomes
KM: Kaplan‐Meier
LASSO: least absolute shrinkage and selection operator
MAF: mutation annotation format
MsigDB: molecular signature database
OS: overall survival
PCA: principal component analysis
RCC: renal cell carcinoma
ROC: receiver operating characteristic
siRNA: small interfering RNA
TCGA: The Cancer Genome Atlas
TCIA: The Cancer Immunome Atlas
TIDE: Tumor Immune Dysfunction and Exclusion
TIIC: Tumor‐infiltrating immune cell
TMB: tumor mutation burden
TME: tumor microenvironment

## 1 INTRODUCTION

Clear cell renal cell carcinoma (ccRCC) is the most common histological subtype of renal cell carcinoma (RCC), accounting for about 75% of all RCC cases [1, 2]. Although ccRCC is frequently diagnosed early, as many as 25%–30% of individuals have distant metastases at initial diagnosis, and approximately 25% experience recurrence after surgical resection [3]. Malignant progression of RCC may lead to inferior vena cava thrombosis [4], with up to 10% of cases accompanied by intravascular tumor thrombus, a potentially lethal complication of RCC [5, 6]. Abnormal coagulation function in ccRCC patients has been significantly linked to distant or lymph node metastases [7, 8]. Recent findings have suggested that coagulation is strongly connected to the tumor microenvironment (TME) [9, 10].

The biological behavior of ccRCC is significantly influenced by the TME, which may also influence how the disease responds to systemic treatment [11]. Previous studies have demonstrated that tumor cells utilize the coagulation system to shape the TME and facilitate distant metastasis [9]. Patients with malignant tumors often suffer from coagulopathy, which is connected to tumor invasion, metastasis, and prognosis [12]. Numerous coagulopathy‐related biomarkers have been recognized as crucial cancer prognostic indicators [13, 14, 15]. Elevated plasma fibrinogen levels in RCC patients have been linked to less favorable clinical outcomes and could be utilized for risk stratification [8]. Anticoagulant therapy has been demonstrated to extend survival time in patients with malignant tumors [16]. Recent studies have indicated that anticoagulation with factor Xa inhibitors could improve the effects of immune checkpoint inhibitors in metastatic malignant melanoma [17]. These observations suggest that coagulation is a crucial factor in the TME and immune escape mechanisms. However, the role of coagulation in ccRCC remains unknown. Therefore, we utilized bioinformatics approaches to examine the interaction of coagulation‐related genes (CRGs) with the TME and their prognostic value for ccRCC patients.

In this study, we utilized an unsupervised clustering algorithm to identify two ccRCC subtypes that are associated with coagulation and have distinct molecular and immune microenvironmental characteristics. The least absolute shrinkage and selection operator (LASSO) method and multivariate Cox regression analysis were then used to construct a coagulation‐related risk score (CRRS) to assess its predictive usefulness in ccRCC. We also investigated how responses to immunotherapy and the immunological microenvironment relate to CRRS. Our results indicated that PLAUR expression was dramatically increased in ccRCC patients who responded to anti‐PD‐1 treatment, indicating its robust predictive value for immunotherapy response. Additionally, elevated PLAUR expression levels were strongly correlated with unfavorable outcomes in ccRCC patients. Suppression of PLAUR resulted in reduced proliferation and migration capabilities of ccRCC. Consequently, using the CRRS model holds promise as an innovative approach for predicting the effect of immunotherapy and the prognosis of individuals with ccRCC.

## 2 MATERIALS AND METHODS

### 2.1 Data collection and processing

The Cancer Genome Atlas (TCGA) website (https://portal.gdc.cancer.gov/) was used to obtain mRNA expression data and clinical details. The E‐MTAB‐1980 cohort was retrieved from the ArrayExpress database (https://www.ebi.ac.uk/arrayexpress/). Gene expression profiles and associated clinical records for RCC were obtained from the Gene Expression Omnibus database (http://www.ncbi.nlm.nih.gov/geo). Using the GSE53757 and GSE126964 cohorts, we investigated the variations in gene transcription between ccRCC samples and normal samples. The whole expression data of ccRCC patients who received anti‐PD‐1 treatment were contained in the GSE67501 cohort. The “sva” package of the R software's ComBat function was employed to fix batch effects [18]. We utilized the “maftools” R package to obtain the mutation annotation format from the TCGA database website [19]. Additionally, the tumor mutation burden (TMB) values of various groups were assessed. A total of 185 CRGs were selected from the molecular signature database (GOBP_NEGATIVE_REGULATION_OF_COAGULATION and GOBP_REGULATION_OF_COAGULATION) and the Kyoto Encyclopedia of Genes and Genomes (KEGG) database (hsa04611). The correlations between all CRGs and patient overall survival (OS) were examined utilizing univariate Cox regression analysis. A *p*‐value cutoff of 0.01 was used to identify the specific genes associated with prognosis in ccRCC patients.

### 2.2 Sample information

The tissue microarray utilized in this study contained 115 ccRCC samples and 58 adjacent normal renal samples acquired from January 2014 to July 2020. The patients' complete clinical information and follow‐up data were retrospectively analyzed in accordance with the RCC staging and grading system [1]. All patients provided informed consent, and the PLA General Hospital's ethics committee approved the study.

### 2.3 Identification of the coagulation subtypes in ccRCC

By employing the “ConsensusClusterPlus” program, we performed a clustering analysis to stratify ccRCC patients into separate molecular subgroups using the expression patterns of prognosis‐related coagulation genes [20]. The stability and quantity of the clusters were assessed using the consensus cluster computation. To assess the effectiveness of clustering, principal component analysis (PCA) was employed. Furthermore, we explored the relationships between various clinicopathological factors to determine the clinical significance of different coagulation subtypes. Additionally, Kaplan‐Meier (KM) survival analysis was conducted to assess the OS among the distinct clusters. Finally, we validated the reproducibility of our findings using the E‐MTAB‐1980 cohort.

### 2.4 Tumor‐infiltrating immune cell (TIIC) analysis

We utilized the CIBERSORT technique to evaluate the proportions of 22 different TIIC types for each specimen in the TME [21]. To comprehensively evaluate the TME, the “ESTIMATE” R package was utilized to compute the stomal score, immunity score, and estimate score for each sample [22].

### 2.5 Functional and pathway enrichment analysis

To study the fundamental biological processes among the various groups, the “GSVA” R package was used to determine the enrichment scores for the 50 hallmark pathways (h.all.v7.5.1 symbols) and KEGG pathways (msigdb.v7.5.1.KEGG.symbols) [23]. Statistically significantly enriched pathways were defined by *p* < 0.05. The overall prognosis of the enriched pathways was also examined using KM analysis.

### 2.6 Construction and verification of the CRRS

To identify the optimal prognostic signature related to coagulation, the TCGA cohort underwent LASSO regression analysis, followed by stepwise multivariate Cox regression analysis. The “glmnet” R package was employed to conduct these analyses. By using these statistical techniques, we aimed to identify the most predictive coagulation‐related variables for prognostic purposes.

To construct the coagulation‐related risk model, the following equation was utilized: risk score = sum of coefficients × expression level of prognostic CRGs.

By applying this method, patients were categorized as high‐risk or low‐risk depending on the model's estimated median risk score. To evaluate the prognostic impact of the risk model, several analyses were conducted. The “survminer” and “timeROC” R programs were applied to generate KM survival curves and receiver operating characteristic (ROC) curves, respectively. The independent prognostic significance of the CRRS was assessed by employing univariate and multivariate Cox regression analyses. Furthermore, the patterns of risk score distribution, mortality situations, and gene transcription variations were also examined. The coagulation‐related prognostic signature was validated using the E‐MTAB‐1980 cohort with the same calculation method. Additionally, the TCGA and E‐MTAB‐1980 cohorts were also used to examine how the CRRSs correlated with other clinicopathological factors.

### 2.7 Immunotherapy and targeted therapy response prediction

This study also sought to investigate if there was a connection between the CRRS and immunotherapy efficacy by analyzing various immunological parameters, including immune checkpoint expression, immunophenoscore (IPS), Tumor Immune Dysfunction and Exclusion (TIDE) score, and TMB scores. The IPS was generated using the Cancer Immunome Atlas (TCIA) (https://tcia.at/home) database [24], while the TIDE score was generated with the TIDE website (http://tide.dfci.harvard.edu/) [25]. Furthermore, TMB estimation was performed for each patient in the TCGA cohort using somatic mutation data. Comparisons were made between patients from the GSE67501 cohort who responded and those who did not respond to anti‐PD1 therapy. Specifically, the gene expression levels of the six genes included in the risk model were examined for potential variations between these two groups. For each ccRCC patient in the TCGA cohort, the semi‐inhibitory concentrations (IC50) often utilized in targeted treatment drugs were predicted using the pRRophetic R package to estimate their therapeutic effectiveness in the high‐ and low‐risk groups [26].

### 2.8 Construction of a nomogram

The “rms” R package was utilized to construct a nomogram, which included ccRCC patient age, gender, clinical stage, and risk score, to predict their clinical prognosis. The accuracy of the nomogram was assessed by comparing its performance in both the TCGA and E‐MTAB‐1980 cohorts. ROC curves were employed to compute the area under the curve (AUC) values and quantify the nomogram's predictive value.

### 2.9 Immunohistochemistry (IHC)

IHC assays were conducted following the protocol outlined in our previous research [27]. An anti‐PLAUR primary antibody (1:100, Proteintech) was used. Three expert pathologists assessed the IHC scoring after staining the ccRCC tissue microarray.

### 2.10 Cell culture and small interfering RNA (siRNA) transfection

A 10% fetal bovine serum supplement was added to the RPMI 1640 medium to culture the ccRCC cell line 786‐O. The 786‐O cell line was obtained from American Type Culture Collection. SiRNAs targeting PLAUR were designed and synthesized (Invitrogen). The sequences of these siRNAs were as follows: si‐PLAUR‐1: 5′‐ CCACUGGAUCCAGGAAGGUGAAGAA‐3′; si‐PLAUR‐2: 5′‐ UUCUUCACCUUCCUGGAUCCAGUGG‐3′. As suggested by the manufacturer, cells were transfected with siRNA using the jetPRIME reagent.

### 2.11 Western blot

The effects of the two siRNAs on PLAUR expression levels were verified using standard western blot analysis. After a 48‐h transfection period, the cells were lysed using radioimmunoprecipitation assay buffer. The PLAUR antibody (10286‐1‐AP) and PD‐L1/CD274 antibody (28076‐1‐AP) were purchased from Proteintech, with an α‐tubulin antibody (diluted 1:1000, EASYBIO, #BE3546) serving as the loading control. The ECL reagent was used to visualize the protein signal.

### 2.12 CCK‐8 assay

Cell proliferation rates were assessed using the CCK‐8 assay in accordance with the manufacturer's provided instructions. Specifically, 786‐O cells transfected with si‐PLAUR‐1 or si‐PLAUR‐2 were seeded in 96‐well plates. The absorbance level at 450 nm was analyzed at various periods (0, 1, 2, and 3 days) after the addition of the CCK‐8 reagent. Each experiment was repeated three times.

### 2.13 Wound healing and migration assays

A wound was generated with a pipette tip after the cells were seeded into a six‐well plate. Pictures of the scratch were taken at 0 and 24 h after the scratch was made. To perform migration experiments, the cells were suspended in a serum‐free medium and placed in the upper chamber of a Transwell (8 μm, Corning) plate. Simultaneously, a complete medium was added to the lower chamber. After 24 h, the cells that moved to the filter's lower surface were preserved, stained, and observed under a microscope. The number of cells was counted using Image J software.

### 2.14 Statistical analysis

All statistical analyses were performed using R software (version 4.2.1). To compare continuous variables among different groups, analysis of variance was conducted. KM survival curves were generated using the “survminer” package to assess differences in OS rates. The prediction effectiveness was evaluated by calculating the AUC value using the “pROC” R package. The results of the univariate and multivariate Cox regression analyses were visually presented using the “forestplot” package. A *p* < 0.05 was used to evaluate statistical significance.

## 3 RESULTS

### 3.1 Identification of two coagulation subtypes in ccRCC

**Figure 1:**
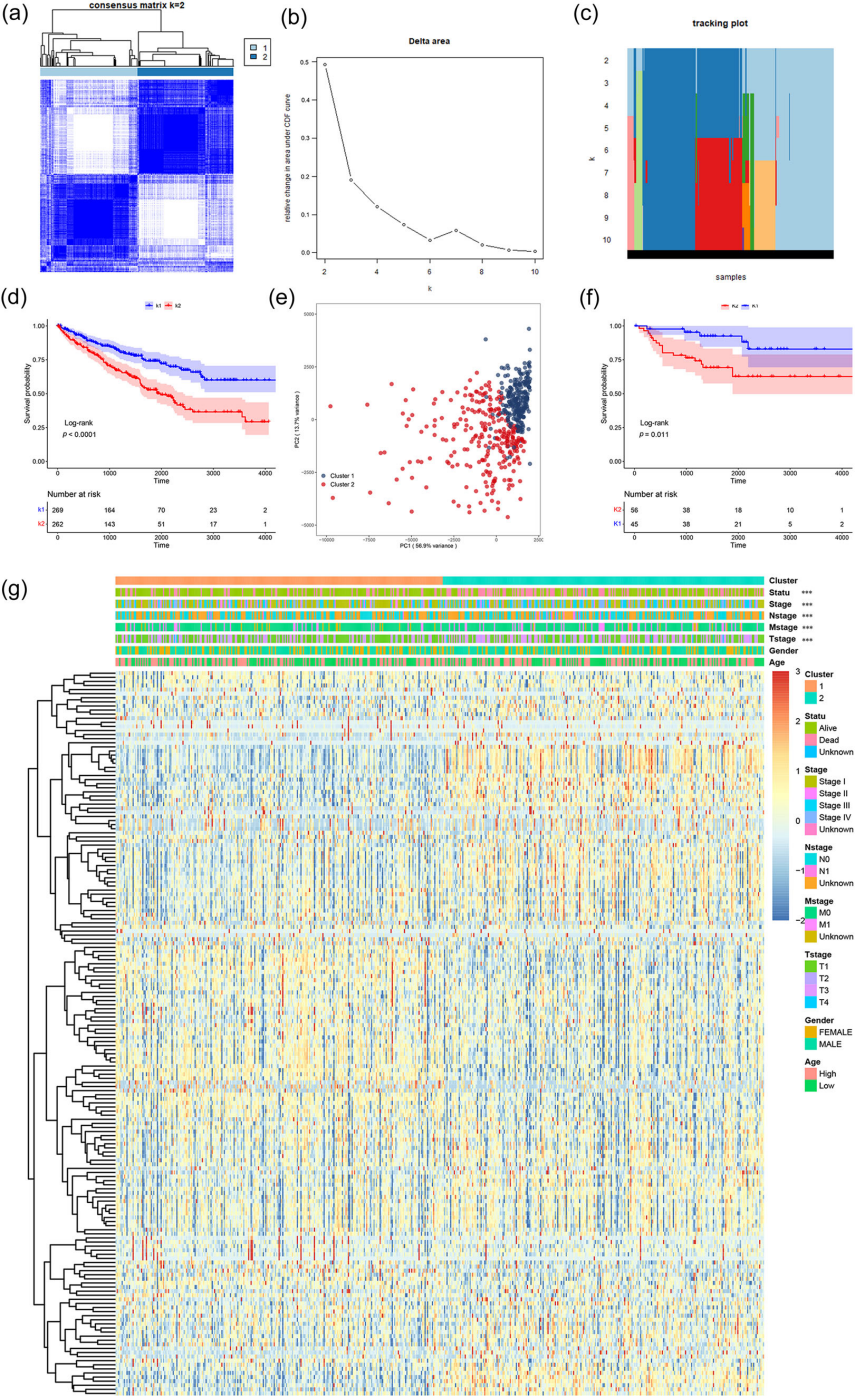
Identification of two coagulation subtypes in clear cell renal cell carcinoma. (a) Patients in The Cancer Genome Atlas (TCGA) cohort were divided into two clusters when k = 2. (b) An area under the cumulative distribution functions curve (k = 2–10). (c) Classify the TCGA cohort into tracking graphs of different subtypes for k = 2–10. (d) Kaplan‐Meier analysis for overall survival (OS) of the two subtypes in the TCGA cohort. (e) Principal component analysis (PCA) of coagulation phenotypes in TCGA cohort. (f) Kaplan‐Meier analysis for OS among the two phenotypes in the E‐MTAB‐1980 cohort. (g) Heatmap of the distribution of clinical parameters of the two subtypes. ***p < 0.001.

In the TCGA cohort, our univariate Cox regression analysis revealed that 58 out of the 185 CRGs exhibited significant correlations with patient survival. These prognostic CRGs were then employed for consensus clustering analysis to determine two different molecular subtypes (Cluster 1, *n* = 269 and Cluster 2, *n* = 262) of ccRCC samples from their expression profiles. Additionally, the unsupervised consensus clustering method with k = 2 as the optimal value was applied to determine the subtypes (Figure 1a–c). The OS of Cluster 2 was considerably lower than that of Cluster 1 according to the KM survival curve (Figure 1d). PCA analysis confirmed remarkable variations between the two phenotypes (Figure 1e). Consensus clustering in the E‐MTAB‐1980 cohort, which similarly displayed two distinct coagulation subtypes with substantial variations in OS across phenotypes, was used to demonstrate the validity of this categorization for ccRCC patients (Figure 1f). We also examined the connection between clinical characteristics and coagulation subtypes in the TCGA cohort. The heatmap clearly distinguished the two subtypes using tumor node metastasis (TNM) and clinical phases (Figure 1g). These results imply that the expression of the CRGs may, via some underlying processes, contribute to the malignant development of ccRCC.

### 3.2 The molecular and immune landscapes of the two coagulation subtypes

**Figure 2:**
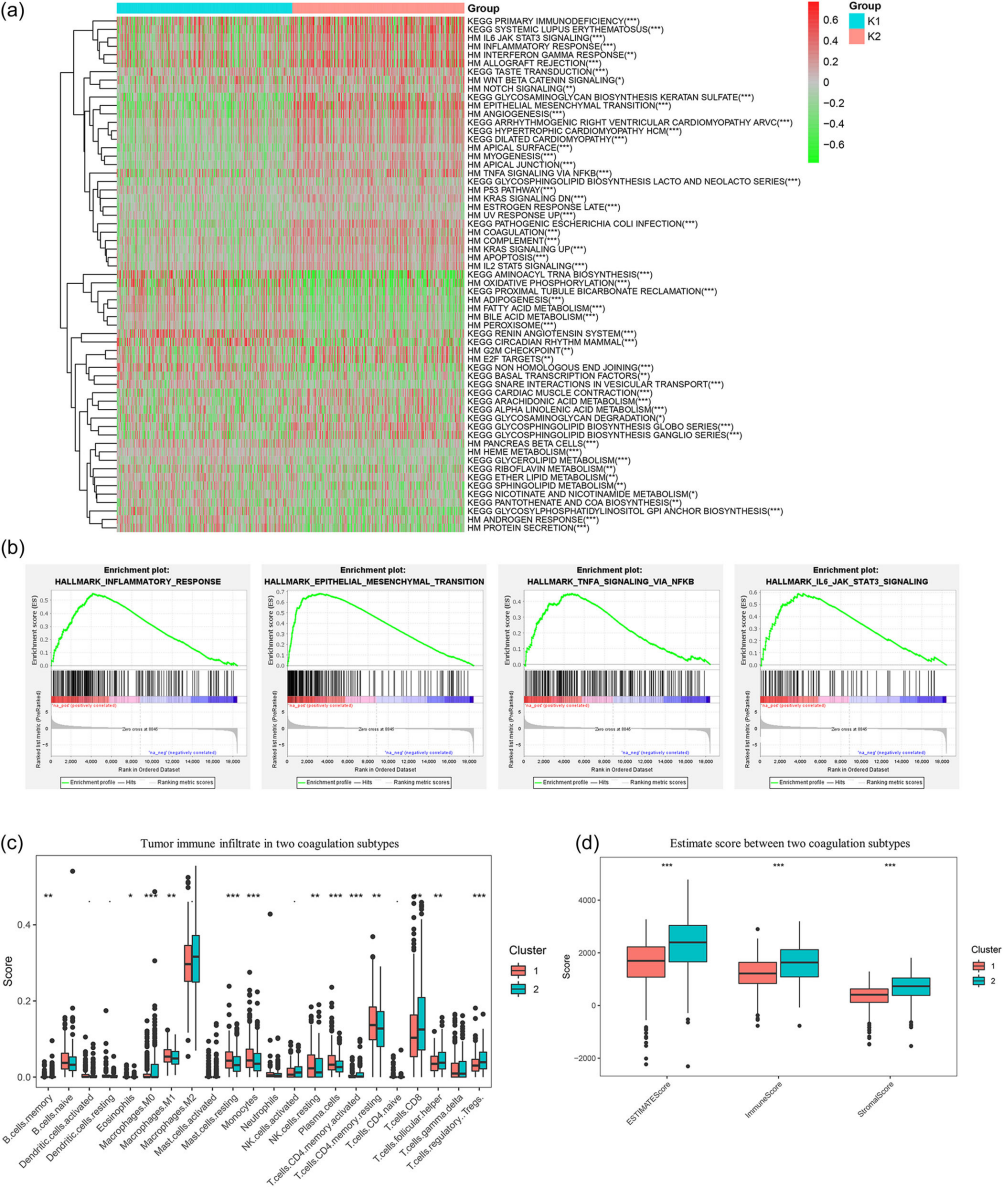
Molecular and immune features of the coagulation subtypes. (a) Heatmap of the biological pathway of two coagulation subtypes. (b) Gene set enrichment analysis demonstrates the pathway of enrichment. (c) Tumor immune infiltrate in two coagulation subtypes. (d) Stromal score, immune score, and estimate score between two coagulation subtypes. *p < 0.05, **p < 0.01, ***p < 0.001.

To explore the underlying molecular mechanisms between the two coagulation subtypes, we conducted gene set variation analysis using hallmark and KEGG pathways. As shown in Figure 2a,b, several pathways, including the epithelial–mesenchymal transition, G2M checkpoint, IL6‐JAK‐STAT3 signaling, tumor necrosis factor α‐signaling‐via‐NFKB, inflammatory response, and apoptosis, had higher activities in Cluster 2. Conversely, the Cluster 1 phenotype displayed an activation of metabolism pathways, including bile acid metabolism, xenobiotic metabolism, drug metabolism cytochrome p450, and fatty acid metabolism. We used the CIBERSORT algorithm with the TCGA cohort to assess the connection between the two coagulation subtypes and immune cell infiltration in the tumor immune microenvironment. Compared with Cluster 2, Cluster 1 had higher infiltration rates of resting mast cells, monocytes, resting natural killer (NK) cells, resting CD4+ memory T cells, plasma cells, and M1 macrophages, while Cluster 2 had significantly higher infiltration rates of resting CD8+T cells, resting follicular helper T (Tfh) cells, and resting regulatory T cells (Tregs) (Figure 2c). Using the ESTIMATE approach, we estimated the immune score, stromal score, and estimate score in the two coagulation subtypes. As illustrated in Figure 2d, samples in Cluster 2 had significantly higher stromal scores, immune scores, and estimate scores compared with those in Cluster 1 (*p* < 0.001).

**Figure 3:**
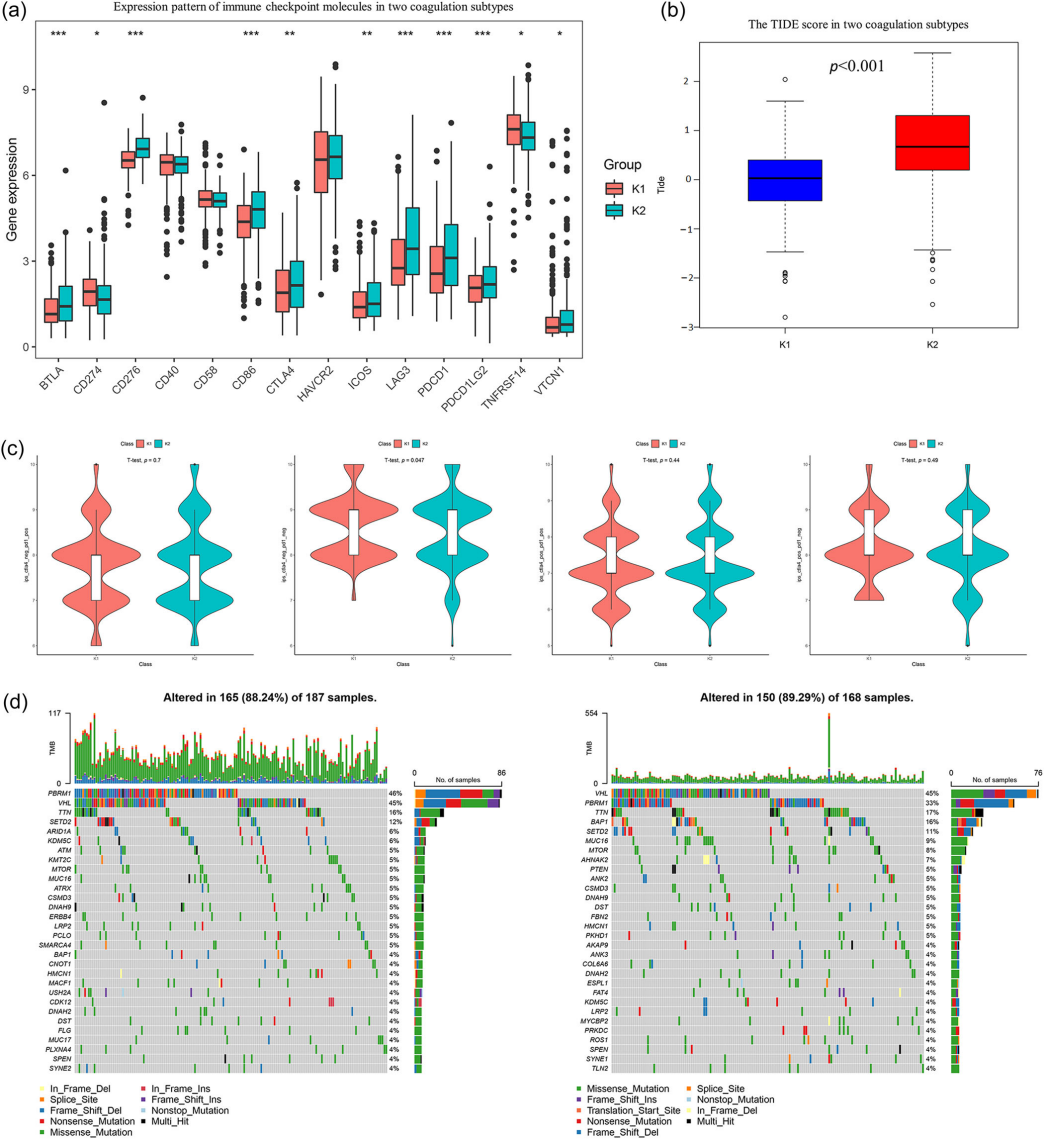
Analysis of immunotherapy response and genomic alterations in two coagulation subtypes. (a) Expression pattern of immune checkpoint molecules in two coagulation subtypes. (b) The Tumor Immune Dysfunction and Exclusion score in two coagulation subtypes. (c) The immunophenoscore (immune cell proportion score) in two coagulation subtypes. (d) Waterfall plot of tumor somatic mutation between the two coagulation subtypes. *p < 0.05, **p < 0.01, ***p < 0.001.

We proceeded to investigate if the coagulation subtypes were significantly linked to the effectiveness of immunotherapy. We analyzed several factors, including the expression of immune checkpoint molecules, TIDE score, and IPS score. The analysis revealed notable variations in immune checkpoint molecule expression levels between the two groups. These included CTLA‐4, CD86, PD‐1, BTLA, LAGS, PD‐L1, ICOS, PD‐L2, TNFRSF14, and VTCN1 (Figure 3a). Moreover, Cluster 2 had a higher TIDE score than Cluster 1 (Figure 3b). We then acquired the IPS score of the TCGA‐KIRC cohort from the TCIA database to more thoroughly assess the variations in immunotherapy. The findings showed that Cluster 1 had a higher IPS score for ctla4_neg_pd1_neg than Cluster 2 (Figure 3c). We also analyzed somatic mutations in the two coagulation subtypes to investigate their immunological characteristics. The mutation rate of Cluster 2 was greater than that of Cluster 1. Figure 3d displays the top 30 genes with the most notable mutation rates in both subgroups, among which PBRM1 (46% vs. 33%) and BAP1 (4% vs. 16%) exhibited the most significant differences between the two coagulation subtypes. These results imply that CRG expression could potentially lead to the malignant development of ccRCC by regulating various biological processes and immune cell infiltration in the TME.

### 3.3 Construction of the CRRS

**Figure 4:**
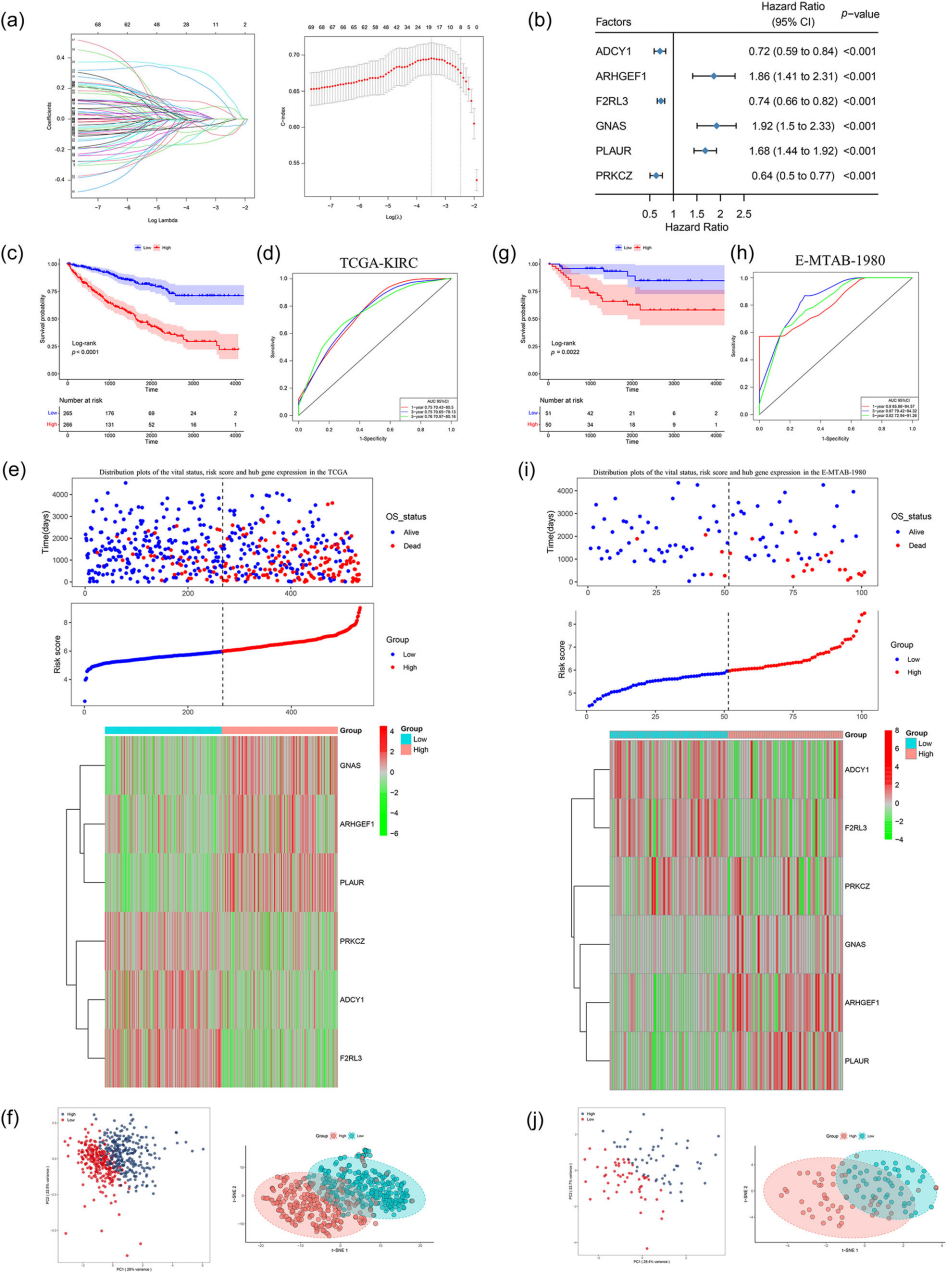
Construction of a coagulation‐related risk score (CRRS) in clear cell renal cell carcinoma. (a) Coefficient profiles of the 58 prognostic coagulation‐related genes and identification of the best parameter (lambda) in the least absolute shrinkage and selection operator regression. (b) Forest plot of six hub genes based on multivariate Cox regression analysis. (c, g) Kaplan‐Meier survival analysis of overall survival (OS) between high‐ and low‐risk groups in The Cancer Genome Atlas (TCGA) and E‐MTAB‐1980 cohorts. (d, h) Receiver operating characteristic curve of CRRS in predicting 1‐, 3‐, and 5‐year OS based on TCGA and E‐MTAB‐1980 cohorts. (e, i) Distribution plots of the risk score, vital status, and the heatmap of gene expressions in the TCGA and E‐MTAB‐1980 cohorts. (f, j) Principal component analysis and t‐distributed stochastic neighbor embedding plot of the TCGA and E‐MTAB‐1980 cohorts.

To evaluate the possible predictive implications of CRGs in ccRCC patients, we utilized LASSO regression and stepwise multivariate Cox regression analyses. These methods were applied in the TCGA cohort to identify the most appropriate model for examining the predictive value of these genes (Figure 4a). Ultimately, we identified six CRGs, including three risk factors (ARHGEF1, GNAS, and PLAUR) and three protective factors (ADCY1, F2RL3, and PRKCZ) (Figure 4b). Using these genes, we established a method to determine the risk score. The risk score was specifically generated via the formula: (−0.16884 × ADCY1) + (0.46689 × ARHGEF1) + (−0.20931 × F2RL3) + (0.50842 × GNAS) + (0.24999 × PLAUR) + (−0.26618 × PRKCZ). The patients were separated into high‐risk and low‐risk groups by utilizing the median value of the estimated risk score as the cut‐off. After generating KM curves, it became clear that the high‐risk group had considerably worse OS than the low‐risk group (Figure 4c). The AUC values for the 1‐, 3‐, and 5‐year OS signatures were 0.75, 0.75, and 0.76, respectively, showing a satisfactory prediction efficiency for the prognostic model in the time‐dependent ROC study (Figure 4d). Additionally, we displayed the TCGA cohort's risk curve, survival state distribution, and risk score heatmap (Figure 4e). We also performed PCA and t‐distributed stochastic neighbor embedding (t‐SNE) analysis, which revealed two distinct groups of patients with different risk levels (Figure 4f). To validate our model, the same procedure was applied to calculate the risk score for each patient in the E‐MTAB‐1980 cohort. The results demonstrated that patients classified into the high‐risk group had notably poorer OS compared with those in the low‐risk group (Figure 4g). Furthermore, using time ROC analysis, we discovered that the AUC values for the CRPS were 0.8 for 1 year, 0.87 for 3 years, and 0.82 for 5 years (Figure 4h). Figure 4i also displays the E‐MTAB‐1980 cohort's risk survival status plots and risk score heatmap. PCA and t‐SNE analysis revealed a distinct trend between the two risk groups, similar to the TCGA cohort (Figure 4j). Overall, our findings suggest that the CRRS is potentially a trustworthy prognostic indicator for ccRCC.

### 3.4 Clinical correlation and risk stratification analysis of CRRS

**Figure 5:**
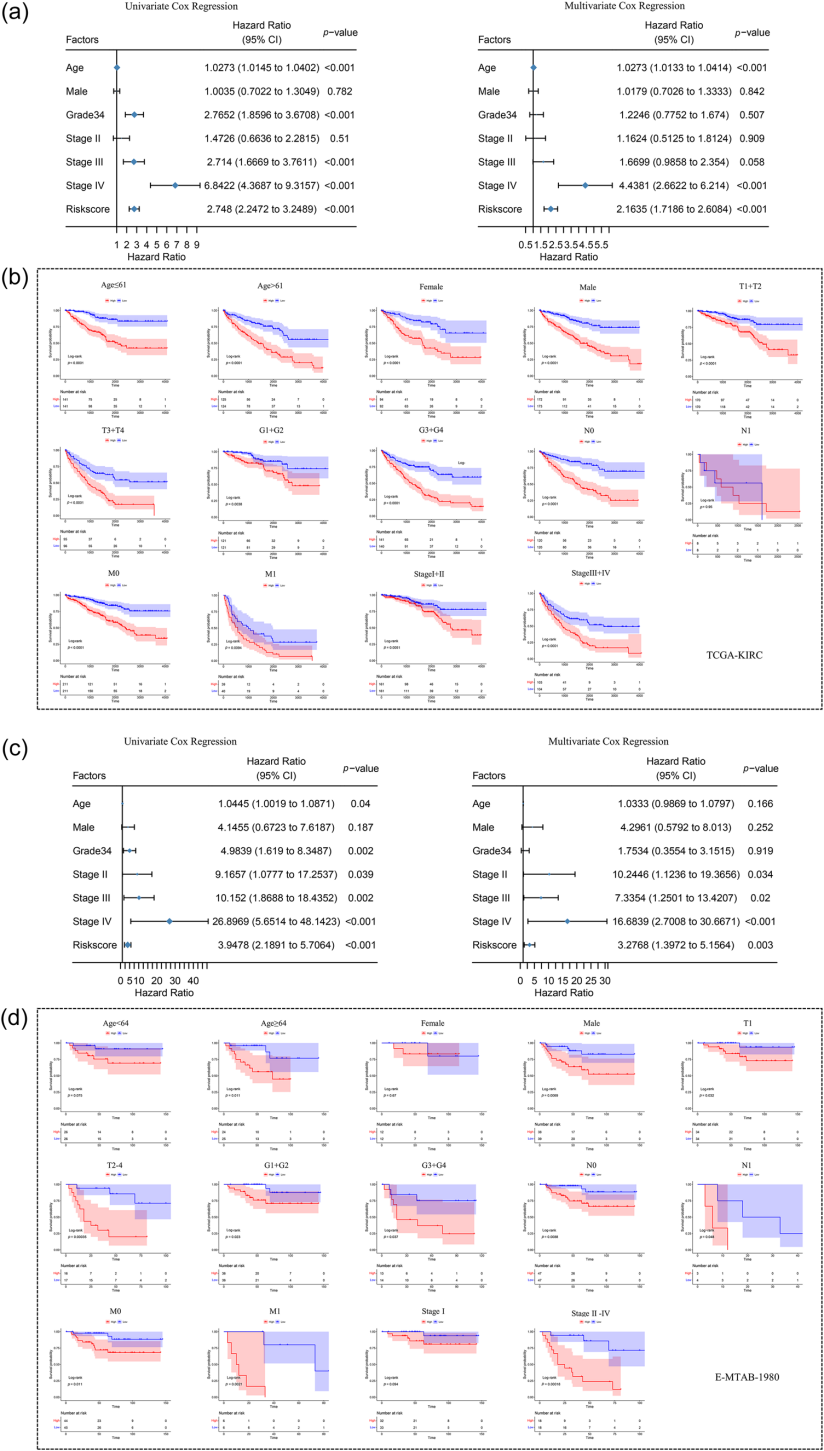
Independent prognostic and stratified analyses based on the risk score in The Cancer Genome Atlas (TCGA) and E‐MTAB‐1980 cohorts. (a, c) Univariate and multivariate Cox regression analysis in the TCGA and E‐MTAB‐1980 cohorts. (b, d) Kaplan‐Meier survival analysis in different clinical subgroups in the TCGA and E‐MTAB‐1980 cohorts.

Furthermore, in both the TCGA and E‐MTAB‐1980 cohorts, we conducted univariate and multivariate Cox regression analyses to assess the predictive value of the risk score alongside other clinical characteristics. The findings suggested that the risk score may be an independent predictive predictor of OS (Figure 5a,c). We additionally examined the associations between the clinical parameters in both cohorts and the risk score. The findings showed a substantial difference in TNM stage, Fuhrman grade, and American Joint Committee on Cancer (AJCC) stage in the TCGA cohort (Supporting Information S1: Figure S1a), indicating that the risk score was strongly related to tumor severity. Moreover, ccRCC patients were separated into two subgroups based on several clinical characteristics to further confirm the clinical significance of the predictive model. Patients with high‐risk scores showed poorer OS in all categories except the pN1 stage subgroup, which had a smaller sample size, as shown by the KM curves (Figure 5b). Patients with advanced tumors in the E‐MTAB‐1980 cohort had greater risk scores than those with early tumor grades, TNM, or AJCC stages (Supporting Information S1: Figure S1b). The KM analyses also revealed notable differences in subgroups, including older age (≥64 years), male, pT2, pN, pM, and stage II–IV (Figure 5d). These results demonstrated that coagulation dysregulation is essential to the emergence and evolution of ccRCC. As a result, the development and progression of ccRCC are significantly influenced by coagulation dysregulation.

### 3.5 Construction of the clinical predictive nomogram in ccRCC

**Figure 6:**
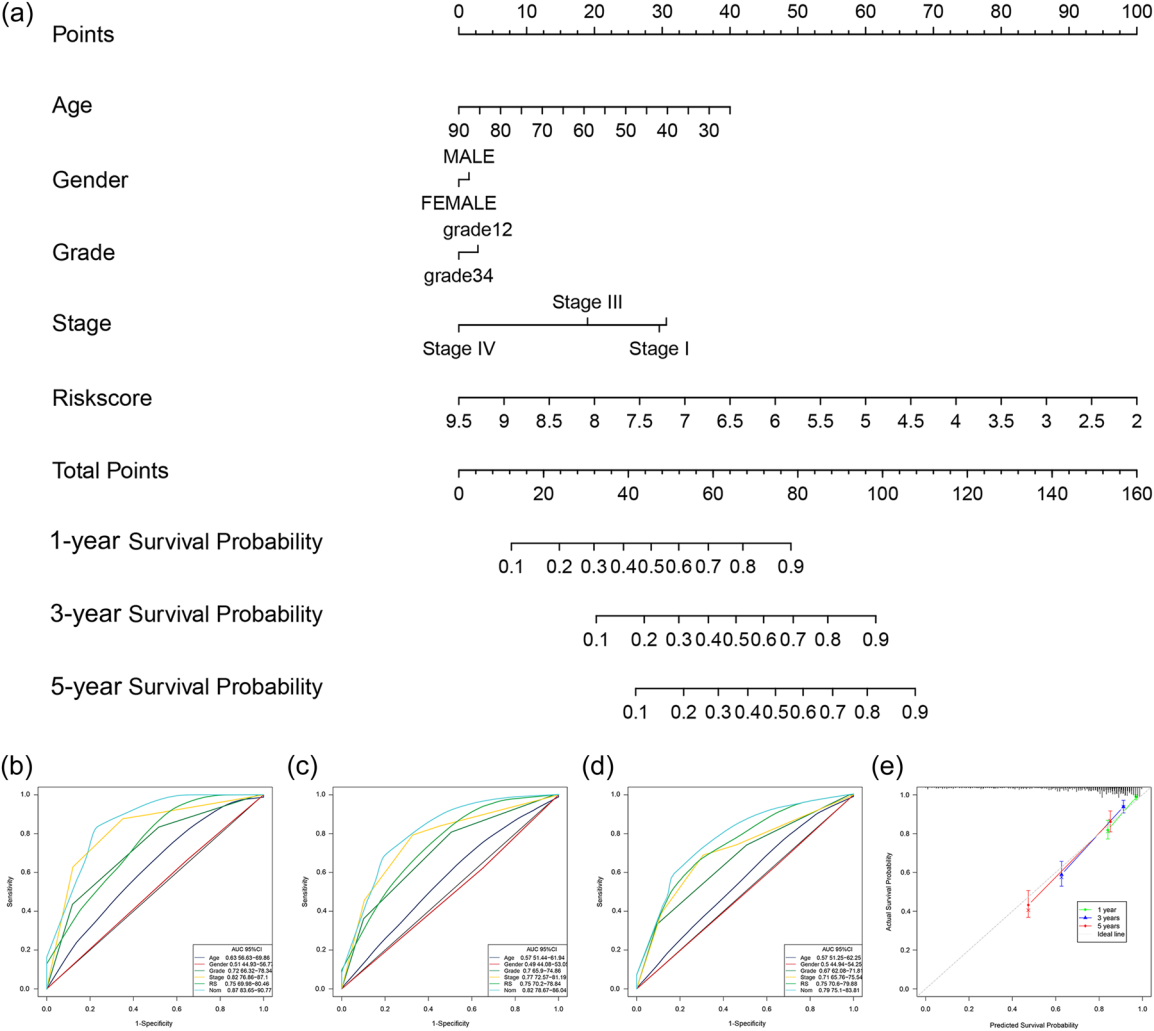
Construction of nomogram in The Cancer Genome Atlas cohort. (a) Nomogram to predict overall survival (OS) at 1, 3, and 5 years based on age, gender, grade, clinical stage, and risk score. (b–d) Receiver operating characteristic curves of the nomogram for predicting 1‐, 3‐ and 5‐year OS. (e) Calibration curve for the nomogram.

Given the substantial relationship of CRRS with malignant development in ccRCC, we developed a personalized nomogram model using age, gender, pathological grade, and clinical stage to help predict OS in the TCGA (Figure 6a) and E‐MTAB‐1980 (Supporting Information S1: Figure S2a) cohorts. The AUC values in the TCGA cohort (0.87, 0.82, and 0.79, respectively) and the E‐MTAB‐1980 cohort (0.92, 0.94, and 0.91, respectively) show that the nomogram executed significantly better performance in predicting 1‐, 3‐, and 5‐year OS than other clinical features (Supporting Information S1: Figure S2b–d). The calibration curves demonstrated a high degree of concordance between the estimated survival rates generated using the nomogram and the actual results in both the TCGA (Figure 6e) and E‐MTAB‐1980 (Supporting Information S1: Figure S2e) cohorts. Overall, our findings show that the nomogram is a potentially valuable tool for clinical management and is a useful model for assessing ccRCC patient prognosis.

### 3.6 Functional enrichment analysis using CRRS

**Figure 7:**
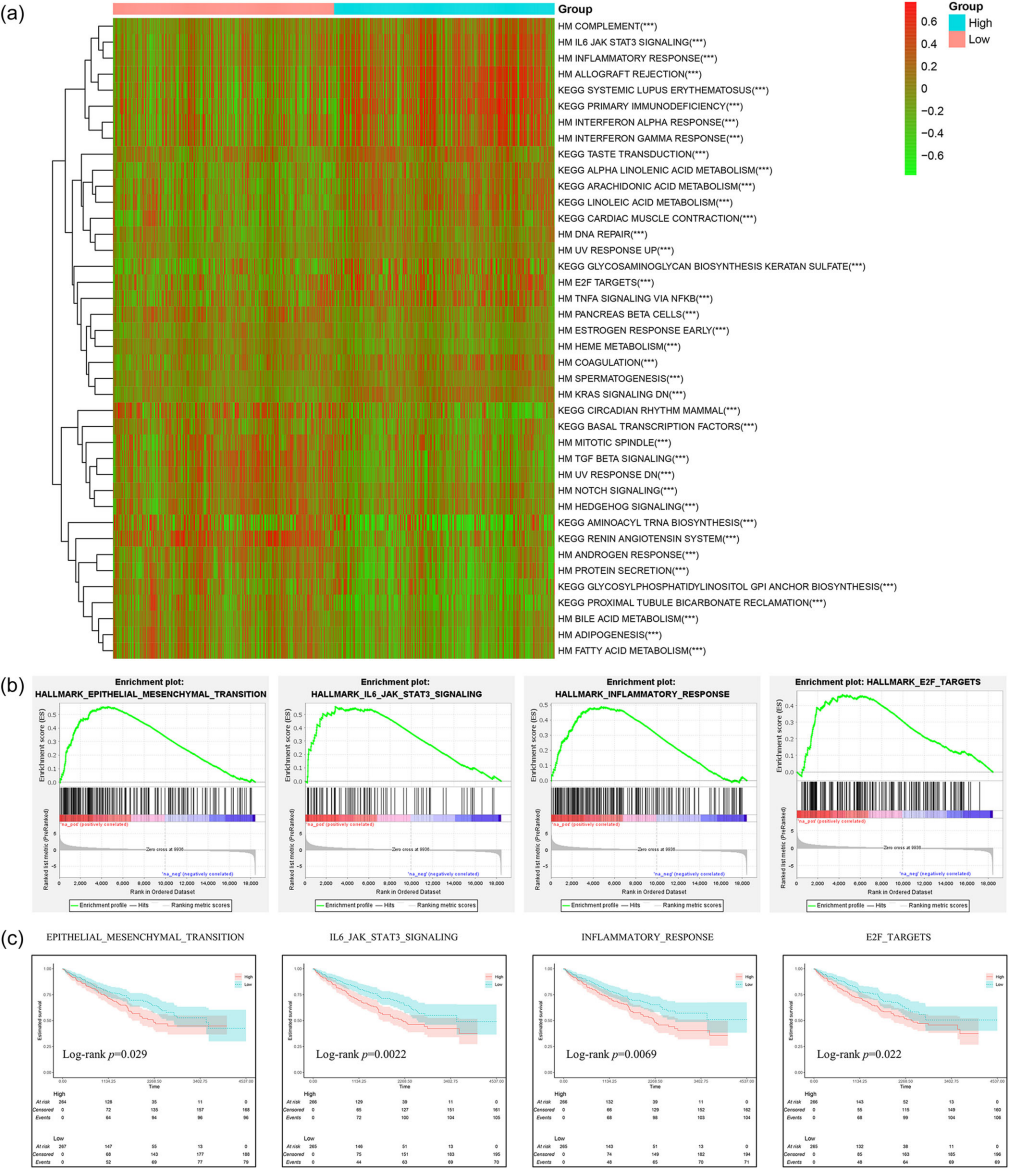
Molecular features of different subgroups of coagulation‐related risk score. (a) Heatmap of the biological pathway of the high‐ and low‐risk groups. (b) Gene Set Enrichment Analysis for high‐ and low‐risk groups. (c) Kaplan‐Meier survival curves of pathways.

We used GSEA‐KEGG and Hallmark analyses to examine variations in pathways between the high‐ and low‐risk groups to further investigate the molecular processes involved with the CRRS. Our findings showed that pathways, such as the epithelial–mesenchymal transition, G2M checkpoint, IL6‐JAK‐STAT3 signaling, inflammatory response, primary immunodeficiency, and E2F targets, were predominantly enriched in the high‐risk group (Figure 7a,b). Using KM survival analysis, we evaluated the prognostic significance of the enriched signature pathways. This analysis revealed differing OS probabilities between the high‐ and low‐risk groups (Figure 7c), explaining the worse prognosis in the high‐risk group.

### 3.7 Analysis of the tumor immune microenvironment and treatment strategy using the CRRS

**Figure 8:**
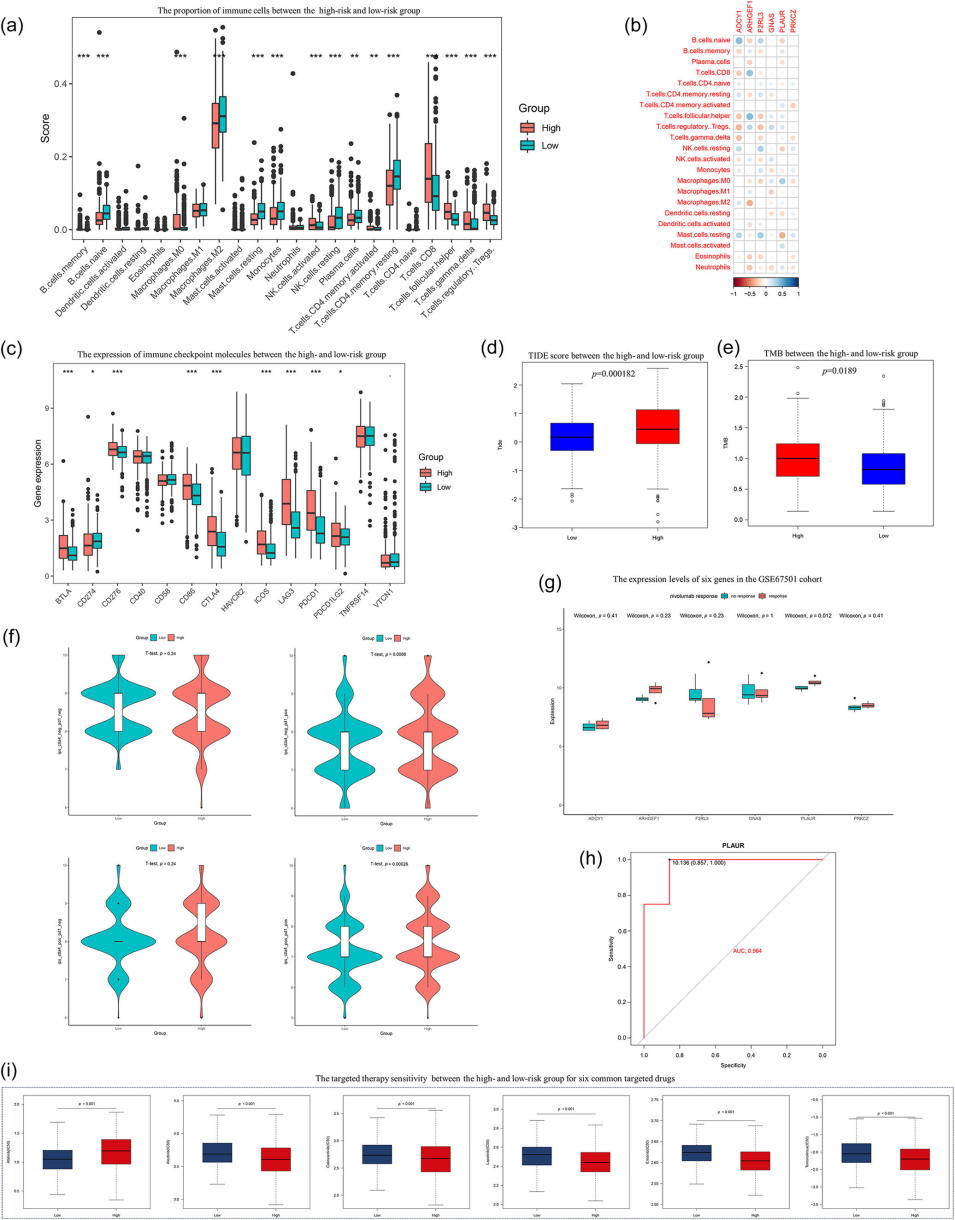
Analysis of tumor immune microenvironment and benefits of immunotherapy and targeted therapy based on the coagulation‐related risk score (CRRS). (a) The proportion of immune cells in high‐ and low‐risk groups. (b) The correlation of six genes in the CRRS with tumor‐infiltrating immune cells. (c) The expression of immune checkpoint molecules in high‐ and low‐risk groups. (d, e) Box plots of the Tumor Immune Dysfunction and Exclusion score and tumor mutation burden between two CRRS groups. (f) The immunophenoscore scores between two CRRS groups. (g) The expression levels of six genes in response and no response groups in the GSE67501 cohort. (h) The area under the curve of PLAUR in the GSE67501cohort. (i) The targeted therapy sensitivity of two CRRS groups for six common targeted drugs. *p < 0.05, **p < 0.01, ***p < 0.001.

For the close connection between coagulation and tumor immunity, we evaluated the differences in immune infiltration between the high‐ and low‐risk groups using the CIBERSORT method. Our findings indicated that the high‐risk group exhibited remarkably higher infiltration of immune cells, including memory B cells, M0 macrophages, activated NK cells, CD8+T cells, Tfh cells, gamma delta T cells, and Tregs. Conversely, the low‐risk group had notably higher levels of resting mast cells, monocytes, naive B cells, M2 macrophages, resting NK cells, plasma cells, and resting memory CD4+T cells (Figure 8a). Additionally, we investigated the relationships between immune cells and the six genes in our suggested model, observing strong correlations (Figure 8b). The correlation of the risk model with immune checkpoint expression levels (Figure 8c) revealed that most immune checkpoint genes (*BTLA*, *CD276*, *CD86*, *CTLA4*, *ICOS*, *LAG3*, and *PD‐1*) were considerably elevated in the high‐risk group. When we used the TIDE algorithm to predict the immunotherapeutic response, a higher TIDE score was observed in the high‐risk group (Figure 8d). We then examined TMB variation between the two risk groups because TMB is strongly associated with immunotherapy efficacy. As anticipated, we found that patients in the high‐risk group had a higher TMB (Figure 8e). Additionally, we utilized TCIA to compare the effects of immunotherapy between the high‐risk and low‐risk groups. For ctla4_neg_pd1_pos and ctla4_pos_pd1_pos, we found that the high‐risk group had greater IPS (*p* < 0.01) than the low‐risk group (Figure 8f). Additionally, we determined the expression levels of the six genes using the ccRCC immunotherapy database (GSE67501). We discovered that PLAUR expression levels were considerably elevated in the group of patients that responded to immunotherapy (Figure 8g). As shown in Figure 8h, the AUC value was 0.964, indicating that PLAUR was a reliable indicator of the therapeutic effects of anti‐PD‐1 treatment. Next, we investigated the relationships between the CRRS and the response to six commonly used targeted drugs (lapatinib, afatinib, erlotinib, alectinib, temsirolimus, and cabozantinib) using the pRRophetic algorithm. Our analysis revealed that patients in the low‐risk group had significantly higher IC50 values for alectinib, cabozantinib, lapatinib, erlotinib, and temsirolimus. In contrast, afatinib had a higher IC50 value in the high‐risk group (Figure 8i). According to these results, CRRS may serve as a novel signature to forecast the clinical effectiveness of immunotherapy and targeted treatment in ccRCC.

### 3.8 PLAUR was associated with ccRCC malignant progression

**Figure 9:**
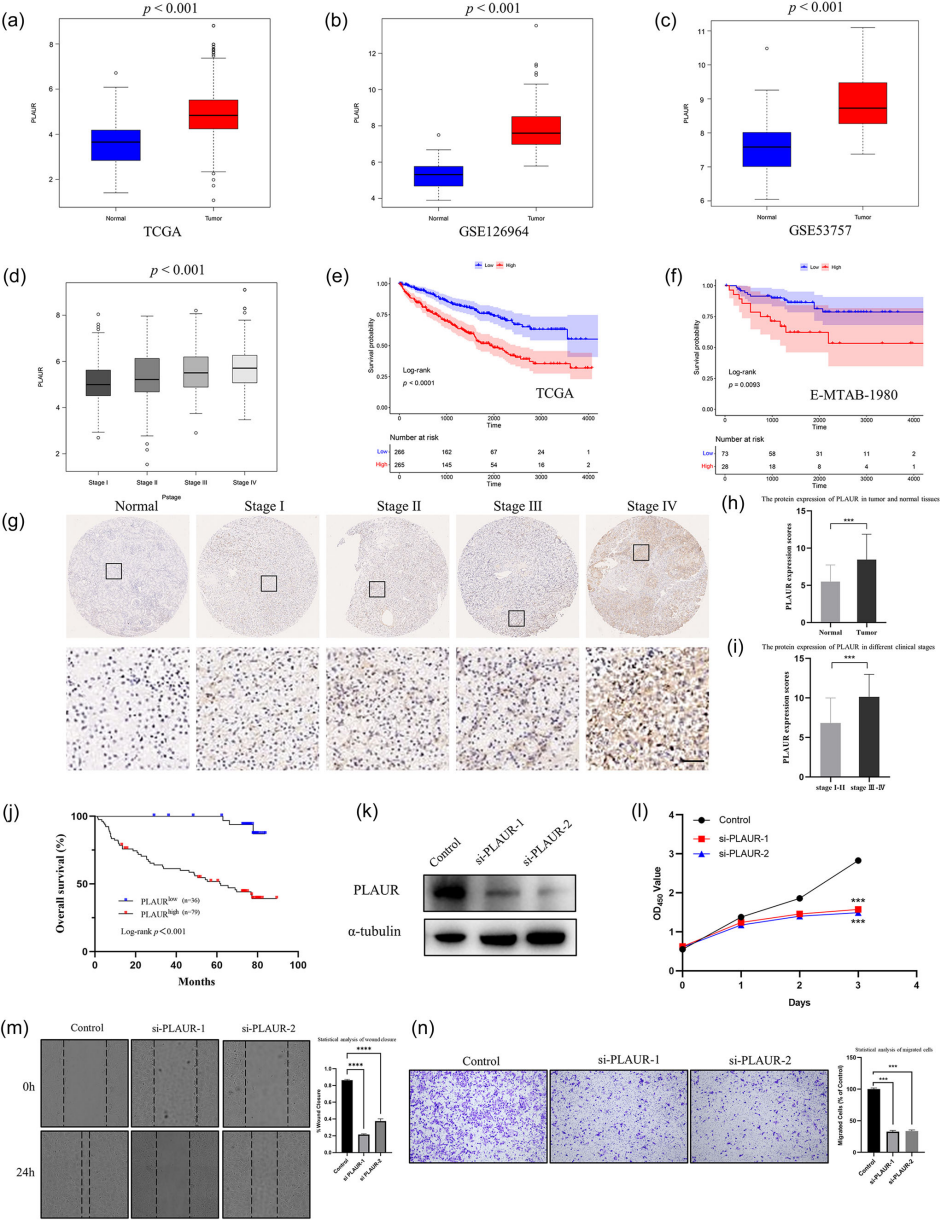
PLAUR was associated with the malignant progression of clear cell renal cell carcinoma. (a–c) The expression of PLAUR in tumor and normal tissues in The Cancer Genome Atlas (TCGA), GSE126964, and GSE53757 data sets. (d) The expression of PLAUR in different clinical stages in the TCGA cohort. (e, f) Kaplan‐Meier survival analysis of PLAUR in the TCGA and E‐MTAB‐1980 cohorts. (g) Immunohistochemical staining of PLAUR in different clinical stages in the tissue microarray (TMA). (h) The protein expression of PLAUR in tumor and normal tissues in the TMA. (i) The protein expression of PLAUR in different clinical stages in the TMA. (j) Kaplan‐Meier survival analysis of PLAUR in the TMA. (k) Western blot analysis confirmed that PLAUR knockdown was effective in 786‐O cells. (l) PLAUR knockdown prevented cell proliferation in 786‐O cells, according to the CCK8 test. (m) A wound healing experiment revealed that PLAUR knockdown reduced the 786‐O cell's capacity for cell migration. (n) Transwell assay results showed that PLAUR attenuation drastically reduced 786‐O cell migration. *p < 0.05, **p < 0.01, ***p < 0.001.

The expression levels of PLAUR and its clinical relevance were then examined in several publicly available data sets and our own tissue microarray data. According to the results of our study, PLAUR expression levels were considerably elevated in renal tumors compared with normal tissues in the TCGA cohort (Figure 9a). Data from two other cohorts (GSE126964 and GSE53757) also supported this conclusion (Figure 9b,c). Furthermore, ccRCC patients in the TCGA cohort showed increased PLAUR expression levels with a clinical stage (Figure 9d). Moreover, high expression of PLAUR in tumor tissues could be used to predict a worse prognosis for patients in both the TCGA and E‐MTAB‐1980 cohorts (Figure 9e,f). We then performed IHC assays on tissue microarrays, including 115 ccRCC and 58 normal renal tissues, to further evaluate PLAUR protein expression. According to our findings, PLAUR protein expression levels were considerably higher in ccRCC tissues (Figure 9g,h) and associated with more advanced clinical stages (Figure 9i). Moreover, KM analysis using a log‐rank test for OS revealed a significant correlation (*p* < 0.001) between higher PLAUR protein expression levels and a poorer prognosis (Figure 9j). Further investigation into the associations between PLAUR expression levels and clinical pathological features revealed that, as shown in Table 1, there were significant associations between PLAUR overexpression and tumor size (*p* = 0.0316), pT stage (*p* = 0.0059), clinical stage (*p* < 0.0001), WHO/ISUP grade (*p* = 0.0018), and metastasis status (*p* < 0.0001).

**Table 1:** The correlation between PLAUR expression and clinicopathological parameters of ccRCC patients.

Variables	No. of patients (*n* = 115)	Low expression	High expression	*p* Value
Age				
≤56	60	17	43	0.4730
>56	55	19	36	
Gender				
Male	94	26	68	0.0745
Female	21	10	11	
Tumor size(cm)				
≤7	73	28	45	0.0316*
>7	42	8	34	
pT stage				
T1+T2	75	30	45	0.0059*
T3+T4	40	6	34	
Clinical stage				
I–II	59	29	30	<0.0001*
III–IV	56	7	49	
WHO/ISUP grade				
1–2	52	24	28	0.0018*
3–4	63	12	51	
Metastasis				
No	62	35	27	<0.0001*
Yes	53	1	52	

*Note*: *p* Value from Chi‐square test.

Abbreviation: ccRCC, clear cell renal cell carcinoma.

* Statistically significant (*p* < 0.05).

Two siRNAs (si‐PLAUR‐1 and si‐PLAUR‐2) were generated to knockdown PLAUR expression in 786‐O cells and further investigate how PLAUR affects the proliferation and migration of renal cancer cells. Western blot analysis was used to validate the knockdown efficiency of each siRNA (Figure 9k). CCK‐8 assays showed that PLAUR knockdown substantially impeded 786‐O cell proliferation (Figure 9l). Moreover, wound healing and Transwell experiments suggested that the downregulation of PLAUR expression levels dramatically decreased 786‐O cell migration (Figure 9m,n). In addition, using the GEPIA website, we observed that PD‐L1 was positively correlated with PLAUR (R = 0.26) (Supporting Information S1: Figure S3a). We examined PD‐L1 protein expression levels after knocking down PLAUR, finding a downward trend of PD‐L1 expression with PLAUR siRNA transfection (Supporting Information S1: Figure S3b). Our findings, therefore, suggested that PLAUR overexpression was related to the ccRCC malignant process and may be considered an indicator for estimating the disease prognosis.

## 4 DISCUSSION

According to previous studies, malignant tumors and the coagulation system are connected in several ways. For example, tumors can activate the coagulation system by producing procoagulant substances, such as tissue factors, cancer procoagulants, angiogenic factors, and cytokines, which can promote thrombin production and fibrin formation. This can in turn support the growth and progression of tumors [28, 29, 30]. Oral anticoagulants are used to avoid such consequences because venous thromboembolic illness is a frequent cause of death in cancer patients [30, 31, 32]. Proliferative cancer cells, platelets, and red blood cells can be detected in venous thrombosis with advanced RCC [5]. The relationship between coagulation, the TME, and renal cancer prognosis is still not fully understood. Therefore, we aimed to provide insights into the roles of coagulation in RCC development, prognostic evaluation, alterations in the tumor immune microenvironment, and immunotherapy response.

In this study, we used several distinct ccRCC data sets for our bioinformatics analysis. The findings demonstrated that biological mechanisms connected to coagulation were crucial for ccRCC development into malignancy. Using the prognostic CRG expression patterns, we categorized ccRCC patients from both the TCGA and E‐MTAB‐1980 cohorts into two distinct coagulation‐related subgroups. Notably, patients belonging to Cluster 2 exhibited a significantly poorer prognosis compared with those in Cluster 1. Patients in Cluster 2 had higher TNM and clinical stages, which further indicated poorer survival in this cohort. Functional enrichment analysis showed that Cluster 2 had significant enrichment of tumor malignant progression and immune‐related signaling pathways. Moreover, there were significant differences in TIIC infiltration rates between the two coagulation subtypes. For example, Tregs and M2 macrophages were significantly increased in Cluster 2. Research has demonstrated that Tregs are immunosuppressive cells that can suppress immunological activation by secreting co‐inhibitory substances or releasing immunosuppressive cytokines [33, 34]. By releasing anti‐inflammatory cytokines, M2 macrophages can hinder tumor immunity and accelerate the growth of malignant tumors [35]. M1 macrophages typically have antitumor effects and can kill tumor cells by releasing various cytotoxic tumor‐killer molecules [35]. The proportion of infiltrated M1 macrophages in Cluster 1 was significantly increased. Therefore, Cluster 2 patients were strongly associated with immunosuppression, which resulted in a poorer prognosis, while Cluster 1 patients were associated with immune activation, which resulted in a better prognosis.

We also discovered six prognostic signatures from the impact of CRGs and distinct coagulation subtypes on ccRCC patient prognosis using LASSO Cox regression and stepwise multivariate Cox regression analyses. By establishing a CRRS, we categorized ccRCC patients into high‐ and low‐risk groups using the median CRRS value as the threshold. Our analysis showed a substantial correlation between CRRS and OS in both the TCGA‐KIRC and E‐MTAB‐1980 cohorts, with patients in the high‐risk group experiencing markedly worse outcomes compared with those in the low‐risk group. Importantly, CRRS emerged as an independent prognostic factor for ccRCC patient prognosis.

Recently, patients with advanced ccRCC have been treated with various immunotherapies, such as combination treatment approaches with anti‐PD1 or anti‐CTLA4 antibodies. However, the majority of patients have not experienced favorable clinical outcomes and immune‐related adverse effects still need to be addressed [36, 37]. Therefore, it is essential to investigate reliable biomarkers that can help predict how immunotherapy affects individuals with RCC. Our research suggests that immune checkpoint molecules, TIDE, TMB, and IPS scores are substantially associated with CRRS, indicating that CRRS may be a crucial indicator of immunotherapy effectiveness. Specifically, PLAUR, a gene within the risk score model, has been identified as a reliable predictor gene in the anti‐PD‐1 RCC data set, with an AUC value of 0.946. In addition, we found that PD‐L1 was positively correlated with PLAUR in RCC, suggesting that PLAUR is closely related to the immune microenvironment. Studies in bladder cancer have shown that PLAUR is positively correlated with the abundance of various immune cells [38]. Glioma samples with high PLAUR expression levels had less CD8+T cell infiltration and more M2 macrophage infiltration, suggesting that PLAUR is a potential marker for predicting macrophage infiltration and the immune microenvironment status in glioma patients [39]. Furthermore, targeted drugs represent a primary strategy for treating RCC. Our findings indicate that the IC50 values of certain targeted drugs significantly vary among different risk groups, indicating that CRRS could help identify ccRCC patients who are potential candidates for targeted therapy.

The PLAUR gene encodes urokinase plasminogen activator receptors and participates in the tumor metastasis process by mediating plasminogen activation and extracellular matrix degradation [40, 41]. PLAUR upregulation has been observed in various malignant tumors, affecting key biological processes including tumor invasion, metastasis, and angiogenesis. These processes are linked to poor patient prognosis [39, 42, 43, 44]. Additionally, aberrant PLAUR expression in tumors is correlated with tumor immunity. As a result, several researchers have used PLAUR as a tumor immunity‐related gene to establish predictive models [45, 46, 47]. In this study, we examined PLAUR expression and its impact on ccRCC patient prognosis using several public data sets. Our findings showed that PLAUR expression levels rise with increasing clinical stage and are significantly higher in ccRCC tissues compared with normal renal tissues. Higher PLAUR expression levels correspond to worse prognoses for ccRCC patients. We also used IHC assays to assess PLAUR protein expression patterns in ccRCC tissue microarrays, which revealed overexpression in tumor tissues that strongly correlated with tumor size, as well as TNM and clinical stages. High PLAUR expression levels predicted poor patient prognosis. Furthermore, siRNA‐mediated knockdown of PLAUR demonstrated notable inhibition of ccRCC cell proliferation and migration.

Our study has some limitations. First, the majority of our research cohorts were derived from public data sets using various sequencing platforms. Thus, the heterogeneity of tumors in these RCC patients cannot be disregarded or eliminated. Second, although we have determined that CRGs play significant roles in the prognosis and immune microenvironment of ccRCC, the underlying biological mechanisms behind these findings remain unclear. Therefore, further prospective research studies and mechanistic investigations are required to clarify the significance of coagulation pathways in RCC.

## 5 CONCLUSION

In summary, we identified two ccRCC coagulation‐related molecular subtypes in patients using CRGs with different prognostic stratification and immune characteristics. Moreover, we established and validated a novel predictive risk model for ccRCC using six CRGs: ARHGEF1, GNAS, PLAUR, ADCY1, F2RL3, and PRKCZ. This risk model could provide therapeutic benefits for immunotherapy and targeted treatment, as well as function as a reliable predictive biomarker. The model may also assist with clinical decision‐making for ccRCC patients. This risk model can possibly help support immunotherapy and targeted treatment regimens by acting as a reliable predictive biomarker. Additionally, it could support ccRCC patient's clinical judgment. Furthermore, our analysis provided further confirmation of the high PLAUR expression levels in ccRCC tissues, which exhibited a significant correlation with an unfavorable prognosis in ccRCC patients. Additionally, our in vitro studies revealed that PLAUR could facilitate ccRCC cell proliferation and migration.

## AUTHOR CONTRIBUTIONS

**Weihao Chen:** Conceptualization (equal); writing—original draft (lead). **Xupeng Zhao:** Formal analysis (equal); methodology (equal); resources (equal). **Yongliang Lu:** Data curation (equal); formal analysis (equal); methodology (equal); resources (equal). **Hanfeng Wang:** Methodology (equal); resources (equal). **Xiyou Wang:** Data curation (equal); resources (equal). **Yi Wang:** Conceptualization (equal); investigation (equal); resources (equal). **Chen Liang:** Project administration (equal); supervision (equal); writing—review and editing (equal). **Zhuomin Jia:** Conceptualization (equal); project administration (equal); supervision (equal); writing—review and editing (equal). **Wei Ma:** Conceptualization (equal); data curation (equal); investigation (equal); methodology (equal); software (lead); supervision (equal); visualization (equal); writing—original draft (equal).

## CONFLICT OF INTEREST STATEMENT

The authors declare no conflict of interest.

## ETHICS STATEMENT

The Ethics Committee of PLA General Hospital approved this study (approval number: S2022‐801‐01).

## INFORMED CONSENT

Written informed consent was obtained from all patients.

## Supporting information

Supporting information.

Supporting information: Figure S1.

Supporting information: Figure S2.

## Data Availability

The TCGA data repository (https://gdc.cancer.gov), ArrayExpress database (https://www.ebi.ac.uk/arrayexpress/), and GEO data repository (https://www.ncbi.nlm.nih.gov/geo) all offer downloads of the data sets used in this investigation. On request, the code is made available.
