# Supplementary material for: Clinical significance, molecular characterization, and immune microenvironment analysis of coagulation‐related genes in clear cell renal cell carcinoma
Source: Cancer Innov. 2024 Jan 7;3(1):e105. doi: 10.1002/cai2.105 (PMC11212306; doi:10.1002/cai2.105)
Supplement: Supplementary file 1 — Supporting information. [file CAI2-3-e105-s001.docx]

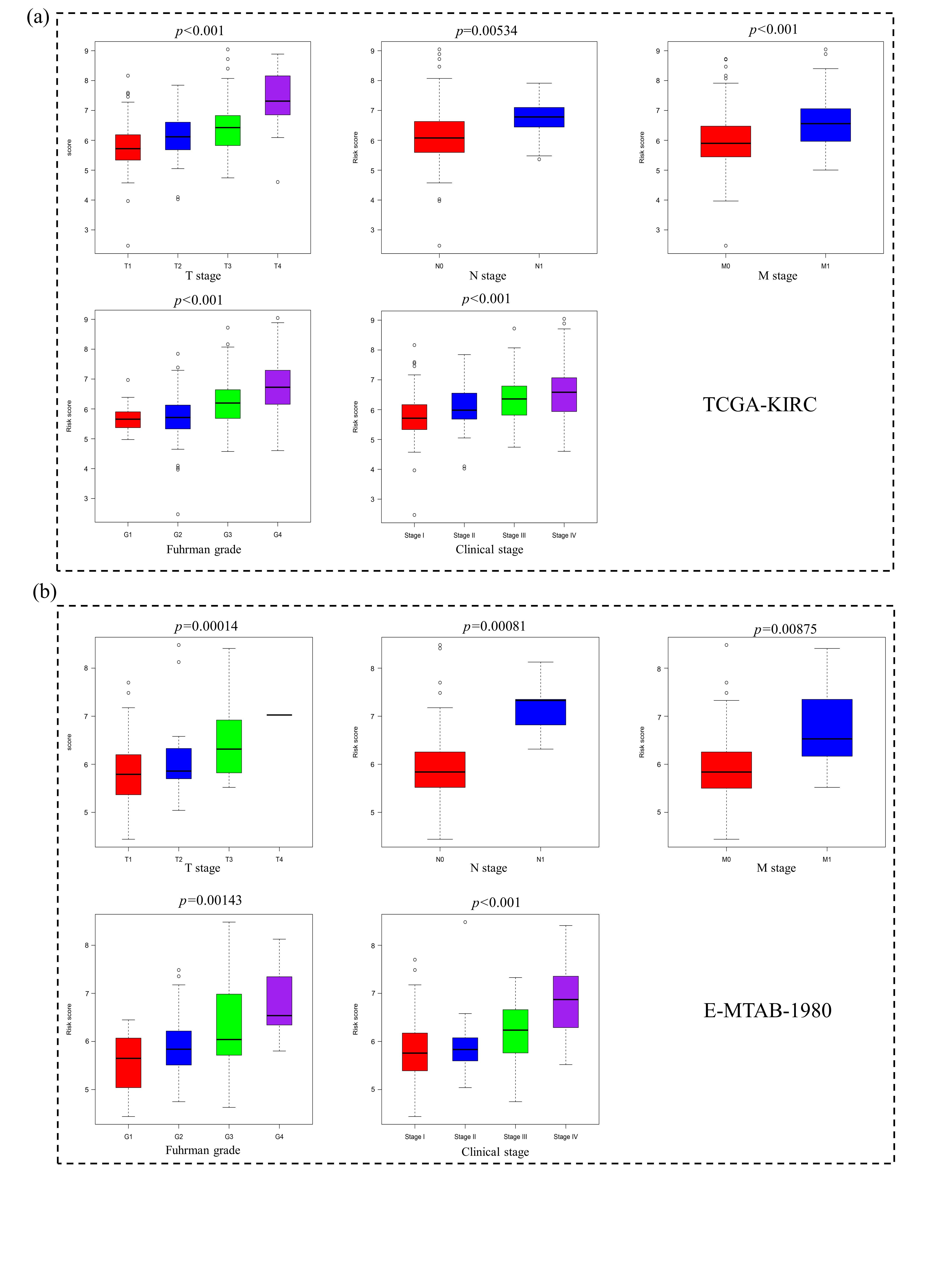


Figure S1. The correlations between clinical features and risk scores in the TCGA and E-MTAB-1980 cohorts. (a, b) Distribution of risk scores in different groups based on clinical features (TNM stage, Fuhrman grade and clinical stage) in the TCGA and E-MTAB-1980 cohorts.


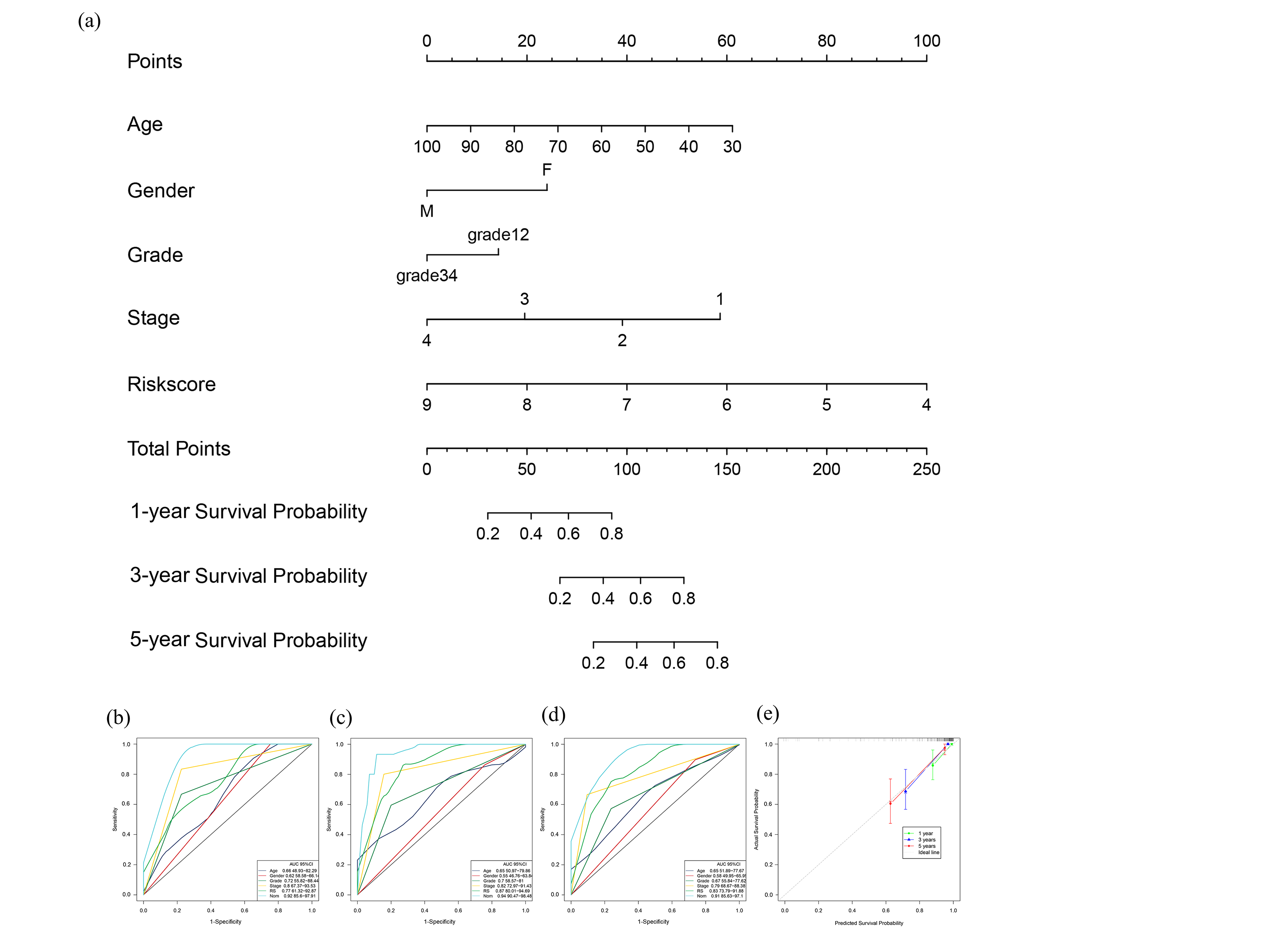


Figure S2. Construction of nomogram in the E-MTAB-1980 cohort. (a) Nomogram to predict overall survival at 1, 3, and 5 years based on the age, gender, grade, clinical stage and risk score. (b–d) ROC curves of nomogram for predicting 1-, 3- and 5-years OS. (e) Calibration curve for the nomogram.


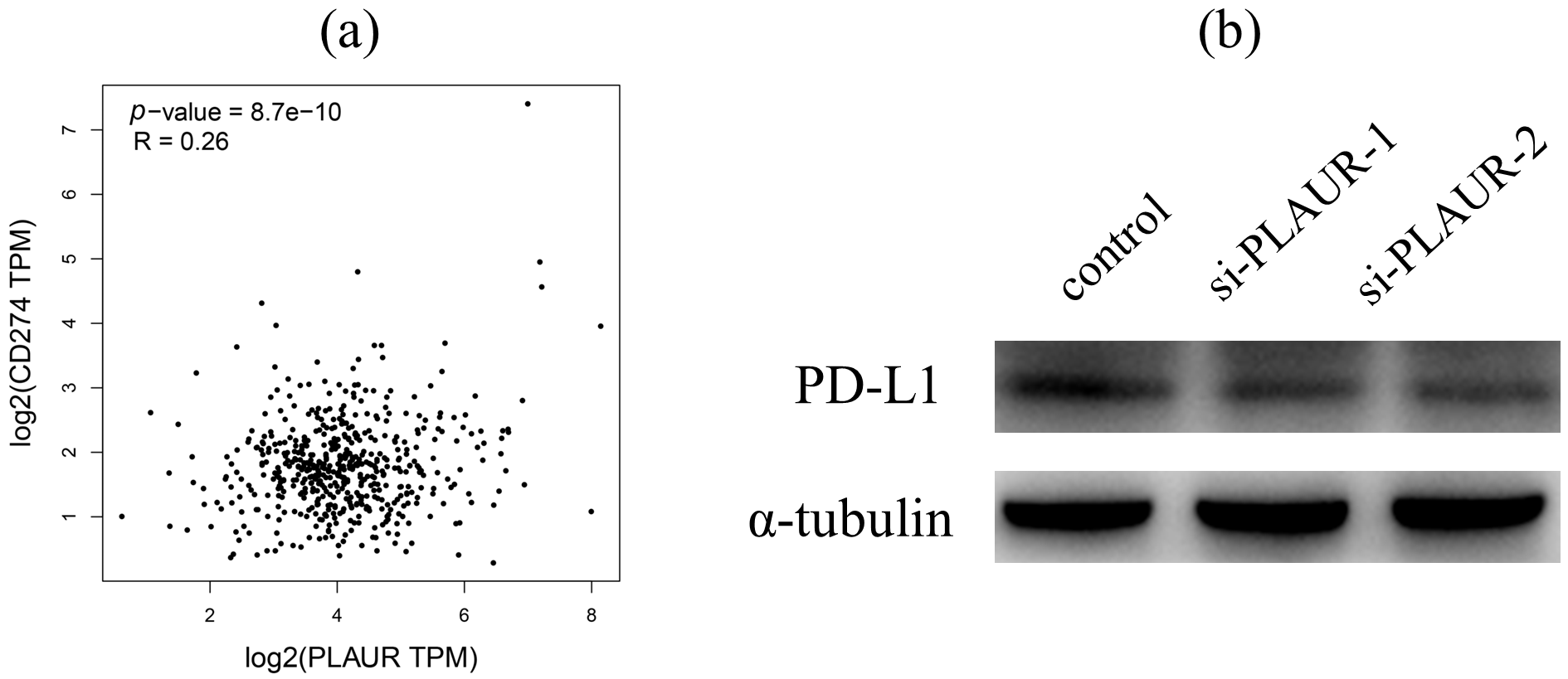


Figure S3. The correlation of PDL1 with PLAUR. (a) The correlation between PLAUR and PDL1 on the GEPIA online website. (b) The expression level of PDL1 after knockdown PLAUR was detected using the Western blot method.
