# Supplementary figures and images for: Clinical significance, molecular characterization, and immune microenvironment analysis of coagulation‐related genes in clear cell renal cell carcinoma
Source: Cancer Innov. 2024 Jan 7;3(1):e105. doi: 10.1002/cai2.105 (PMC11212306; doi:10.1002/cai2.105)

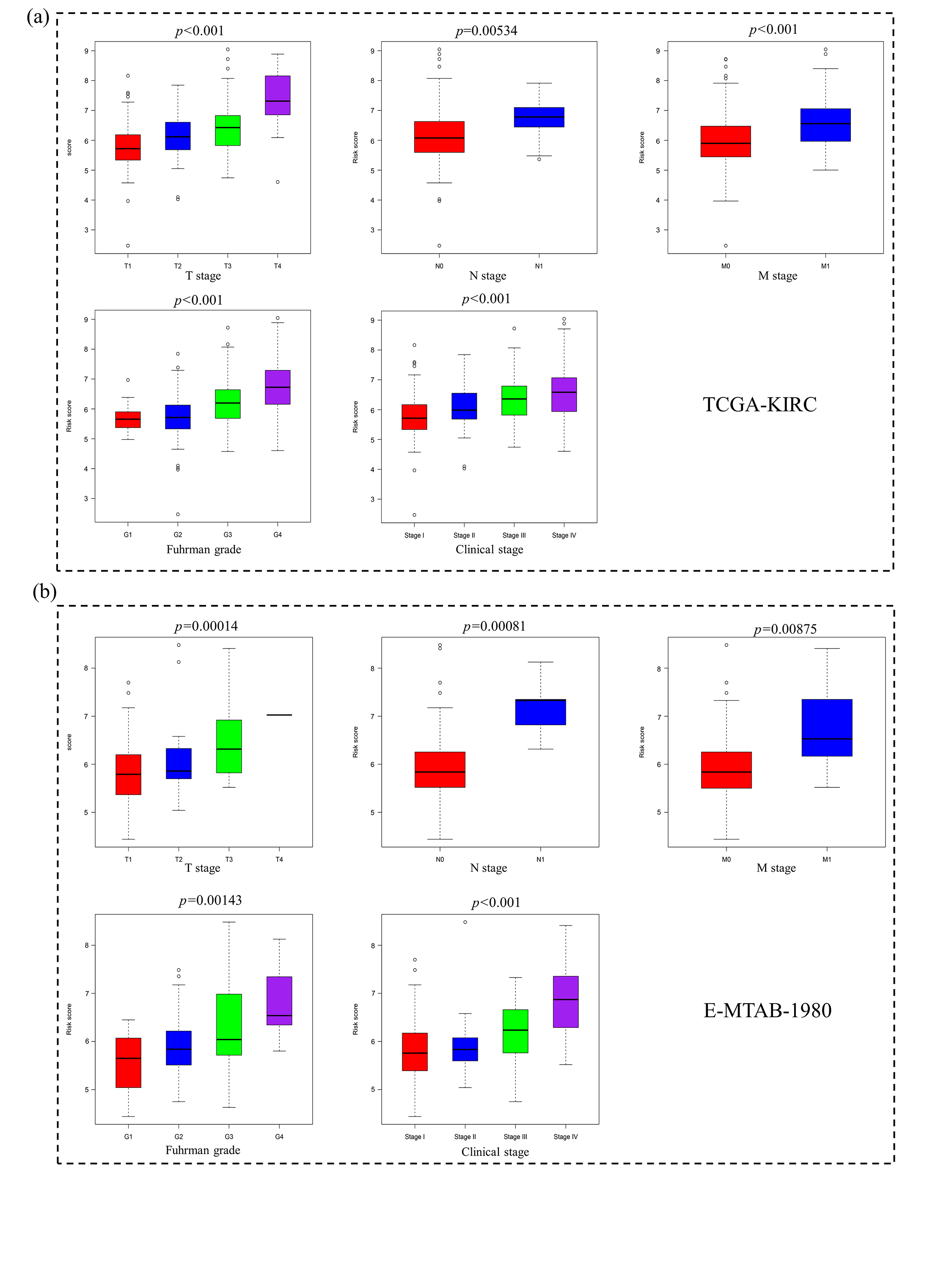

Supplement: Supplementary file 2 — Supporting information: Figure S1. [file CAI2-3-e105-s002.tif]

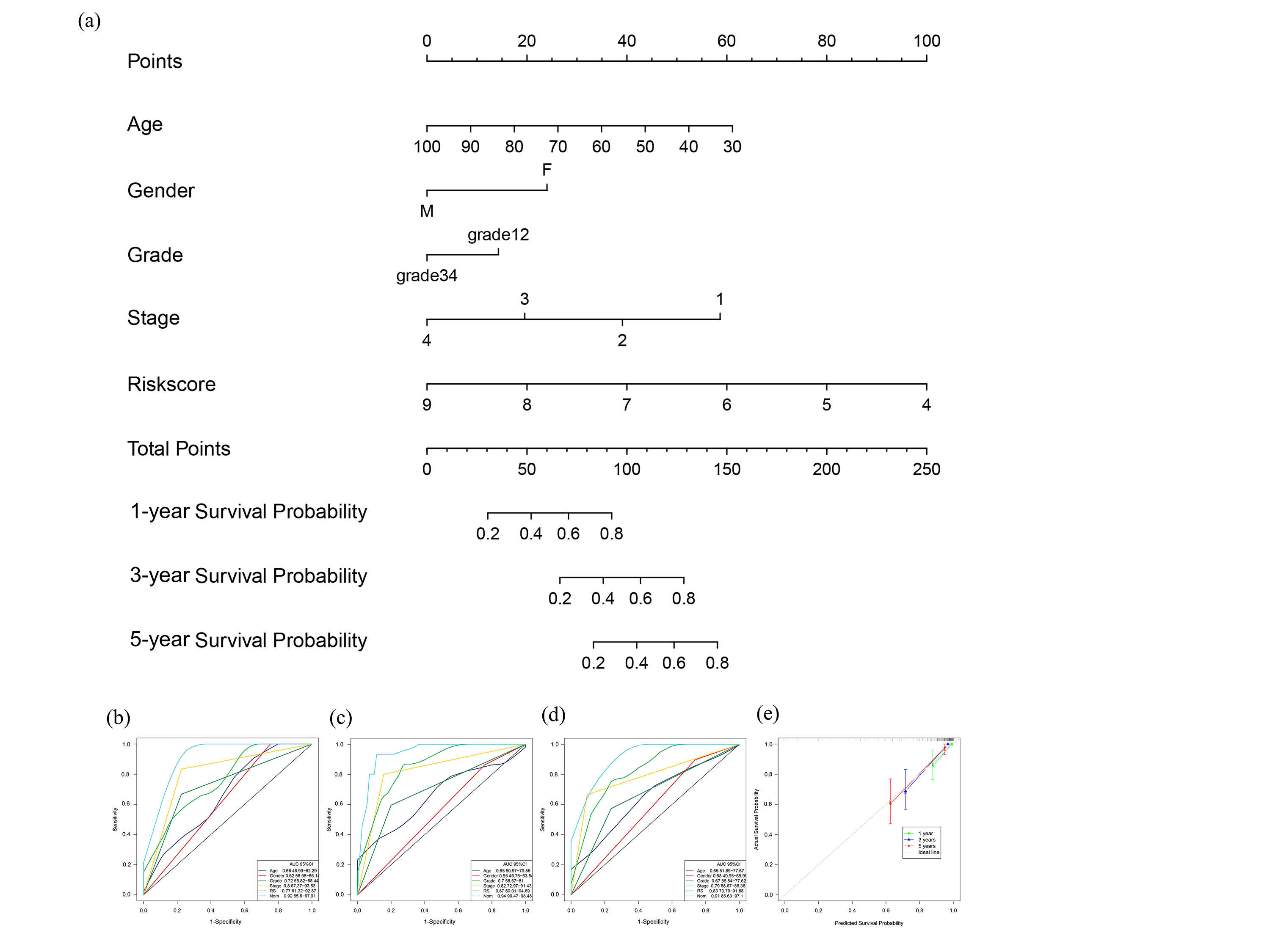

Supplement: Supplementary file 3 — Supporting information: Figure S2. [file CAI2-3-e105-s003.tif]
